# Sustained increase of paediatric invasive *Streptococcus pyogenes* infections dominated by M1_UK_ and diverse *emm*12 isolates, Portugal, September 2022 to May 2023

**DOI:** 10.2807/1560-7917.ES.2023.28.36.2300427

**Published:** 2023-09-07

**Authors:** Catarina Gouveia, Maria Paula Bajanca-Lavado, Rafael Mamede, Ana Araújo Carvalho, Fernanda Rodrigues, José Melo-Cristino, Mario Ramirez, Ana Friães, Portuguese Group for the Study of Streptococcal Infections, Portuguese Study Group of Pediatric Invasive Streptococcal Disease, Portuguese Group for the Study of Streptococcal Infections, Margarida Pinto, Miguel Seruca, João Marques, Isabel Peres, Teresa Pina, Isabel Lourenço, Teresa Ferreira, Cristina Marcelo, Isabel Daniel, Odete Chantre, Teresa Vaz, Marília Gião, Rui Ferreira, Rui Tomé Ribeiro, Celeste Pontes, Luísa Boaventura, Catarina Chaves, Teresa Reis, Henrique Oliveira, Catarina Chaves, Mariana Silva, Ana Aguiar, Hugo Loureiro, Adriana Pedrosa, Hermínia Costa, Maria Fátima Silva, Maria Amélia Afonso, Mariana Fardilha, Natália Novais, Isabel Brito, Luís Marques Lito, Ana Bruschy Fonseca, Filomena Martins, Maria Ana Pessanha, Elsa Gonçalves, Teresa Morais, Cristina Toscano, Paulo Lopes, Angelina Lameirão, Gabriela Abreu, Aurélia Selaru, Ana Paula Mota Vieira, Margarida Tomaz, Cláudia Ferreira, Marta Nicolau, Maria Helena Ramos, Ana Paula Castro, Virgínia Lopes, Fernando Fonseca, Ana Paula Castro, Nuno Canhoto, Teresa Afonso, Ilse Fontes, Paulo Martinho, Gina Marrão, Ana Domingos, José Grossinho, Manuela Ribeiro, Helena Gonçalves, Alberta Faustino, Maria Cármen Iglesias, Maria Paula Pinheiro, Rui Semedo, Adriana Coutinho, Luísa Gonçalves, Olga Neto, Luísa Sancho, José Diogo, Filipa Fortunato, Isabel Nascimento, Nadiya Kruptsala, Cláudia Fidalgo, Elmano Ramalheira, Raquel Diaz, Sónia Ferreia, Inês Cravo Roxo, Isabel Vale, Maria João Tomás, Maria Antónia Read, Valquíria Alves, Margarida Monteiro, Margarida Rodrigues, José Mota Freitas, Sandra Vieira, Elsa Calado, Paula Pinto, Ana Custódio, Maria Favila Menezes, José Germano de Sousa, Mariana Bettencourt Viana, Marvin Oliveira, Isaura Terra, Vitória Rodrigues, Sofia Marques, Joana Selada, Patrícia Pereira, Jesuína Duarte, Paula Pinto, Ezequiel Moreira, Adília Vicente, Fátima Vale, Joana Ramos, Rita Gralha, Ana Helena Correia, Paula Gama, Catarina Silva-Costa, Joana Gomes-Silva, Marcos Pinho, Célia Rodrigues Bettencourt, Miguel Pinto, Portuguese Study Group of Paediatric Invasive Streptococcal Disease, Sónia Aires, Eurico Gaspar, Manuela Ferreira, Fernanda Pereira, Graça Pombo, Maria José Dinis, Paulo Teixeira, José Amorim, Cláudia Monteiro, Diana Moreira, Sofia Arosa, Laura Marques, Margarida Tavares, Maria Manuel Zarcos, Sílvia Almeida, Fernanda Rodrigues, Jorge Rodrigues, Pedro Carvalho, Catarina Gouveia, Ana Isabel Carvalho, Alexandra Costa, Elsa Gonçalves, Filipa Prata, João Calado Nunes, Julieta Morais, Florbela Cunha, Paula Correia, Ana Margarida Chaves, Sofia Lima, João Neves, João Bivar, Pedro Flores, Sofia Fraga, Isabel Brito, Cristina Didelet, Estela Veiga, Carla Cruz, Graça Seves, Céu Novais, Maria João Virtuoso, Nancy Guerreiro, Francisco Gomes, Dora Gomes, Carolina Gonçalves, Nuno Canhoto

**Affiliations:** 1 Infectious Diseases Unit, Pediatric Department, Hospital de Dona Estefânia, Centro Hospitalar Universitário de Lisboa Central, Lisbon, Portugal; 2 Laboratório Nacional de Referência a Infeções Respiratórias a Agentes Bacterianos, Departamento de Doenças Infeciosas, Instituto Nacional de Saúde Dr. Ricardo Jorge, Lisbon, Portugal; 3 Instituto de Microbiologia, Instituto de Medicina Molecular, Faculdade de Medicina, Universidade de Lisboa, Lisbon, Portugal; 4 Hospital Pediátrico, Centro Hospitalar e Universitário de Coimbra, Coimbra, Portugal; 5 The members of the networks are listed under Collaborators

**Keywords:** *Streptococcus pyogenes*, group A streptococcus, paediatric infections, epidemiology, M1UK, *emm* type, whole genome sequencing

## Abstract

Since autumn 2022, observed numbers of paediatric invasive group A *Streptococcus* infections in Portugal (n = 89) were higher than in pre-COVID-19 seasons. Between September 2022 and May 2023, the dominant diagnoses were pneumonia (25/79), mostly with empyema (20/25), and sepsis (22/79). A number of cases required admission to intensive care (27/79) and surgery (35/79), and the case fatality rate was 5.1% (4/79). Genomic sequencing (n = 55) revealed multiple genetic lineages, dominated by the M1_UK_ sublineage (26/55) and more diverse *emm*12 isolates (12/55).

In line with reports from several other European countries [1], since late 2022, an increase of paediatric (aged < 18 years) invasive group A *Streptococcus* infections (piGAS) has been notified in the context of an ongoing prospective surveillance of piGAS in Portugal, including a high prevalence of pneumonia with empyema. Here, we report the epidemiological and clinical characteristics of the infections between September 2022 and May 2023, as well as the main molecular characteristics and antimicrobial resistance of the *S. pyogenes* (Lancefield group A* Streptococcus* (GAS)) isolates.

## Prospective surveillance of paediatric invasive group A *Streptococcus* infections in Portugal

A nationwide prospective surveillance of piGAS in Portugal has been ongoing since January 2014. All paediatric departments are invited to notify piGAS with demographic data, clinical diagnosis and outcome, while clinical microbiology laboratories are requested to submit all GAS recovered from normally sterile sites for antimicrobial susceptibility testing and genomic sequencing. Confirmed and probable piGAS are defined according to recent guidelines [2].

**Figure 1:**
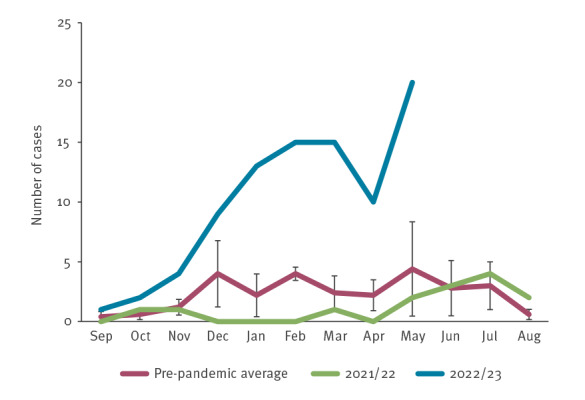
Monthly distribution of cases with paediatric invasive group A *Streptococcus* infections, Portugal, 2014/15–2022/23

After a considerable decrease recorded during the seasons of 2019/20 to 2021/22 (September to August), piGAS in Portugal started to rise again in May 2022. From December 2022 to February 2023 there was a notable increase, and numbers thereafter remained much higher than the average of pre-pandemic years (Figure 1).

The pre-pandemic average comprises the seasons from 2014/15 to 2018/19, is represented in red with 95% confidence interval error bars. The 2021/22 season is represented in green and the 2022/23 season in blue. Seasons are considered as September to August of the following year.

Overall, between 1 September 2022 and 31 May 2023, 89 piGAS cases were recorded (85 were confirmed and four were probable), which is 4 times higher than the average for the same period in pre-pandemic seasons (2014/15 to 2018/19, mean: 21.4 cases, range: 16–26), and at least 2.5 times the number of cases in any of the previous complete seasons (mean: 20.8 cases, range: 1–36 cases). The median age of the patients was 3 years (interquartile range (IQR): 2–6); 45 were males (50.6%) and 44 were females (49.4%).

## Clinical presentations and outcomes

Clinical information was collected through a questionnaire included in the notification form, and was available for 79 cases (Table). In line with the overall cohort, the median age was 3 years (IQR: 2–6 years) and there were 43 males (54.4%) and 36 females (45.6%). Most cases occurred in children without underlying medical conditions (68/79; 86.1%), but varicella (17/69; 24.6%) or upper respiratory infection (16/65; 24.6%) within 2 weeks before admission was frequently reported, although not associated with specific clinical presentations.

**Table:** Characteristics of cases with paediatric invasive group A *Streptococcus* infections, Portugal, 1 September 2022–31 May 2023 (n = 79)

Characteristics	piGAS cases	
	n	%
**Underlying medical condition**		
Yes^a^	11	13.9
No	68	86.1
**Infection within 2 weeks before hospital admission** ^b^		
Varicella	17/69	24.6
Respiratory infection	16/65	24.6
**Presentation**		
Rash	33	41.8
Pharyngitis	28	35.4
Vomiting	17	21.5
Diarrhoea	11	13.9
**Diagnosis**		
Pneumonia	25	31.6
Pneumonia with empyema	20	25.3
Sepsis	22	27.8
Bacteraemia without focus	19	24.1
Bone and joint infection	17	21.5
STSS	14	17.7
Meningitis	6	7.6
Necrotising fasciitis	1	1.3
**Complications**		
Coagulopathy	10	12.7
Renal failure	10	12.7
Multiple organ dysfunction syndrome	9	11.4
Respiratory failure	7	8.9
Hepatic dysfunction	5	6.3
Thrombosis	4	5.1
**Management/outcome**		
Adjunctive clindamycin	55	69.6
IVIG treatment	8	10.1
Surgery or drainage^c^	35	44.3
PICU admission	27	34.2
Sequelae at 30 days	10	12.7
Case fatality rate	4	5.1

IVIG: intravenous immunoglobulin; PICU: paediatric intensive care unit; STSS: streptococcal toxic shock syndrome.

^a ^Underlying medical conditions included chronic lung or neurological diseases, chronic skin conditions and sickle cell disease. The exact diseases or conditions were not specified in the questionnaire.

^b^ Infections occurring within the 2 weeks preceding the hospital admission or present at admission. Information on preceding varicella infection was available for 69 cases and on preceding respiratory infection for 65 cases. No data was obtained on the detection of specific respiratory viruses.

^c^ Mostly joint (13/35; 37.1%) and pleural fluid drainage (12/35; 34.3%).

The median overall length of hospital stay was 10 days (IQR: 5–17). Paediatric intensive care unit (PICU) admission (27/79; 34.2%) and surgical interventions or drainage (35/79; 44.3%) were frequent.

Three patients died within the first 24 h of admission with streptococcal toxic shock syndrome (STSS), and one died at day 15 with complicated meningitis. Sequelae at 30 days were noted in 10/79 cases (12.7%), although the type and severity of sequelae were not directly included in our questionnaire and were not systematically reported. The reported sequelae included foot ischaemia requiring amputation in a child with STSS who had both *S. pyogenes* and *S. pneumoniae* identified by PCR in the pleural fluid, and mild limb paresis in a child with a cerebral abscess. Four patients with pneumonia still had respiratory symptoms at 30 days follow-up.

## Bacterial characteristics

Of the 85 confirmed cases, 13 were diagnosed by real-time PCR on culture-negative samples (11 from pleural fluid and 1 from a fasciitis deep tissue sample). Bacterial isolates were available for 55 of the 72 culture-positive cases.

**Figure 2:**
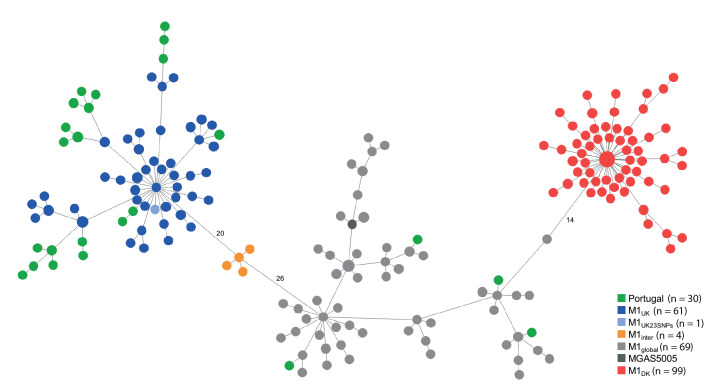
Minimum spanning tree of paediatric invasive *emm*1 group A *Streptococcus* isolates, Portugal, 1 September 2022–31 May 2023 (n = 30), and of *emm*1 isolates from United Kingdom, 2009–2016 (n = 135) and Denmark, 2022–2023 (n = 99)

**Figure 3:**
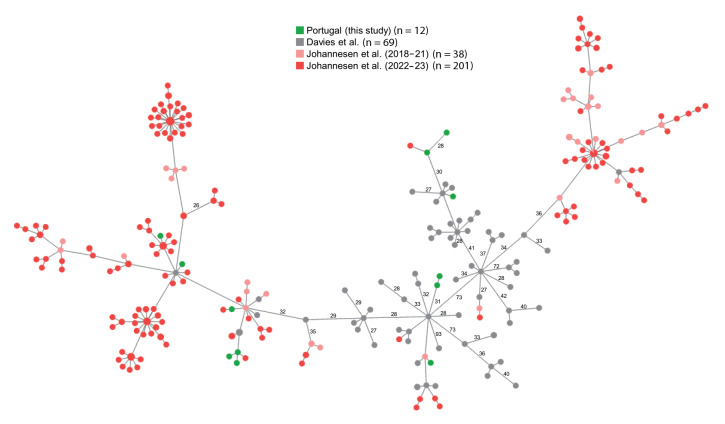
Minimum spanning tree of paediatric invasive *emm*12 group A *Streptococcus* isolates, Portugal, 1 September 2022–31 May 2023 (n = 12), and of *emm*12 isolates from Denmark, 2018–2023 (n = 239) and a global collection, 2001–2015 (n = 69)

Illumina sequencing was performed (data available in the European Nucleotide Archive (ENA); accession number PRJEB65018), and *emm* types and seven gene multilocus sequence types (STs) were retrieved from de novo assemblies [3] (detailed high-throughput sequencing and data analysis methods can be found in the Supplementary materials). The lineages defined by *emm*1-ST28/1319 (n = 30) and *emm*12-ST36/242 (n = 12) together accounted for 76.4% of the isolates (the *emm* types and STs of each isolate can be found in Supplementary Table S1). Screening for the characteristic 27 M1_UK _sublineage SNPs (the number of SNPs found in each isolate is available in  Supplementary Table S1) [4] and core genome multilocus sequence typing (cgMLST) analysis [3] support the classification of 26/30 *emm*1 isolates as M1_UK_ (Figure 2). The gene encoding superantigen SpeC was identified in two *emm*1 isolates (data available in Supplementary Table S1), both belonging to the M1_UK_ sublineage and not to the recently described *speC*-positive M1_DK_ sublineage [5], which was not found among our isolates (Figure 2). The cgMLST of the *emm*12 isolates, analysed together with a dataset of *emm*12 genomes selected to represent the global diversity of this *emm* type [6] and with the recently published *emm*12 genomes from Denmark [5], reveals a high genetic diversity, with no clear dominance of any sublineage (Figure 3).

cgMLST: core genome multilocus sequence typing; UK: United Kingdom.

The tree was generated with the cgMLST profiles of paediatric invasive *emm*1 isolates recovered in Portugal, 1 September 2022–31 May 2023 (n = 30; green), non-invasive isolates (n = 135) recovered in London, UK [4] carrying 27 (dark blue), 23 (light blue), 13 (orange) or 0 (light grey) of 27 SNPs characteristic of the M1_UK_ sublineage, invasive and non-invasive isolates from Denmark (n = 99) [5] carrying the 15 SNPs characteristic of the M1_DK_ sublineage (red), and the M1 reference strain MGAS5005 (dark grey). The list of genomes used is available in Supplementary Tables S1 and S2. The size of each node is proportional to the number of isolates with that particular cgMLST profile on a logarithmic scale. Link distances separating the previously identified sublineages are labelled as the number of allelic differences between nodes (from a total of 1,249 compared loci). All isolates from Portugal carrying ≥ 26 M1_UK_ SNPs (n = 26, found in Supplementary Table S1) grouped with the M1_UK_ isolates, while the four isolates without M1_UK_ SNPs grouped with M1_global_ isolates. Link distances in the minimum spanning tree vary from 1 to 16 allelic differences between M1_UK_ nodes, from 2 to 4 allelic differences between M1_inter_ nodes, from 1 to 6 allelic differences between M1_DK_ nodes, and from 1 to 40 allelic differences between M1_global_ nodes. A maximum likelihood tree of the same isolates can be found in Supplementary Figure S1. Detailed high-throughput sequencing and data analysis methods can be found in the Supplementary materials.

The tree was generated with the cgMLST profiles of paediatric invasive *emm*12 isolates recovered in Portugal, 1 September 2022–31 May 2023 (n = 12; green), genetically diverse invasive and non-invasive *emm*12 isolates (n = 69) from a global collection [6] (grey), and invasive and non-invasive *emm*12 isolates (n = 239) recovered in Denmark during 2018–21 (pink) or 2022–23 (red). The list of genomes used is available in Supplementary Tables S1 and S2. Link distances ≥ 26 allelic differences are labelled (from a total of 1,128 compared loci). A maximum likelihood tree of the same isolates can be found in Supplementary Figure S2. Detailed high-throughput sequencing and data analysis methods can be found in the Supplementary materials.

Among 48 cases with molecular and clinical information, PICU admission was more frequent among patients with *emm*1 (12/28 cases (43%) vs 2/20 cases with other *emm* types (10%), Fisher’s exact test, p = 0.02). There were no other significant associations of *emm*1 with clinical presentation or outcome, although all three deaths occurred in patients with this *emm* type.

Antimicrobial resistance was tested in all available isolates by disc diffusion according to EUCAST guidelines [7] (the antimicrobial susceptibility profile of each isolate can be found in Supplementary Table S1). Only tetracycline (n = 1) and norfloxacin (n = 2) resistance were identified; the first associated with the presence of the *tet*(M) gene while the latter with the S79F and A121V mutations in the *parC* gene.

## Discussion

Following a period of very low levels of invasive GAS infections during the COVID-19 pandemic, a marked increase has been reported in multiple European countries and in some regions of the United States (US) [1,5,8-15]. In most cases, this increase was particularly striking in paediatric age groups and was associated with a shift in the dominant clinical presentations towards pulmonary infections with empyema [5,8-11,13,16]. Pneumonia, often complicated with pleural effusion, was the most frequent diagnosis among our patients. The case fatality rate (5.1%) recorded up to May 2023 is comparable to that reported in other studies [5,9,13].

Remarkably, this surge has not occurred simultaneously in all reporting countries. In the Netherlands, piGAS has increased since early 2022 [8], while in England, France, Ireland, Denmark, Spain and the US, the increase was noted in the autumn/winter of the same year [5,9-11,13,14]. In Portugal, the most dramatic increase relative to pre-pandemic seasons occurred from January 2023 onwards, with the number of cases remaining persistently high until May, despite a dip in April. This contrasts with the previous seasons, where mostly single-month peaks were observed. The reasons underlying the differences in upsurge timing between different countries remain unclear. The non-pharmaceutical interventions during the COVID-19 pandemic and their detrimental impact on child immunity, associated with the increased circulation of respiratory viruses, have been suggested as potential drivers of this multinational upsurge of piGAS [8,9]. Several countries reported a temporal coincidence between the piGAS surge and the respective respiratory viral season, in particular for respiratory syncytial virus (RSV) and influenza, or a high prevalence of preceding/concurrent viral infection among piGAS cases [9,10,13,14]. In Portugal, national data of all-age surveillance of viral respiratory infections indicate that the circulation of influenza and RSV was most intense between late October 2022 and early January 2023 [17], therefore preceding, but not coinciding, with the majority of piGAS cases. Only 24.6% of cases reported a respiratory infection within the 2 weeks preceding hospitalisation with piGAS, further questioning a major role for these infections in driving the piGAS surge in Portugal.

As observed in other countries [5,8-10,13], multiple genetic lineages were identified, despite the clear dominance of the M1_UK_ sublineage (47.3%). The association between *emm*1 and PICU admission observed in Portugal and Denmark [5] supports an increased virulence of lineages expressing this *emm* type, which remains significantly associated with invasive disease [5,18]. The genomic analysis of our *emm*12 isolates (21.8%) revealed a much higher genetic diversity when compared with *emm*1, with no apparent expansion of any sublineage. Given that *emm*12 was significantly associated with non-invasive infections in Denmark [5], its abundance in piGAS during 2022/23 in Portugal may reflect its high prevalence in the population.

The absence of macrolide and lincosamide resistance among piGAS in 2022/23 follows the decreasing trend recorded among all-age invasive and pharyngeal infections in Portugal [19,20] and is in line with the low prevalence of macrolide resistance reported during the surge in England [15].

## Conclusion

Portugal is experiencing an exceptionally pronounced and long surge of piGAS, with no signs of substantial reduction in spring of 2023, despite a brief, unexplained decrease in April. Therefore, GAS should remain one of the main suspected aetiological agents in children presenting with compatible clinical signs. The presence of pharyngitis or rash may be important clues for suspecting this agent. Together with data from other countries, our genomic analysis indicates that multiple sublineages are important in the post-COVID-19 multinational surge of piGAS.

## Supplementary Data

1260000 Supplementary materials and figures

## Supplementary Data

55000 Supplementary tables
