# Supplementary material for: Sustained increase of paediatric invasive Streptococcus pyogenes infections dominated by M1UK and diverse emm12 isolates, Portugal, September 2022 to May 2023
Source: Euro Surveill. 2023 Sep 7;28(36):2300427. doi: 10.2807/1560-7917.ES.2023.28.36.2300427 (PMC10486195; doi:10.2807/1560-7917.ES.2023.28.36.2300427)
Supplement: Supplementary materials and figures [file 23-00427_FRIAES_Supplement.pdf]

Supplementary Material

This supplementary material is hosted by *Eurosurveillance* as supporting information alongside the article "Sustained multi-month increase of paediatric invasive *Streptococcus pyogenes* infections dominated by $M1_{UK}$ and diverse *emm*12 isolates in Portugal, 2022/23", on behalf of the authors, who remain responsible for the accuracy and appropriateness of the content. The same standards for ethics, copyright, attributions and permissions as for the article apply. Supplements are not edited by *Eurosurveillance* and the journal is not responsible for the maintenance of any links or email addresses provided therein.

Materials and Methods

Bacterial strains and data sets

A set of 55 bacterial isolates of paediatric invasive group A streptococcus (GAS) collected in Portugal between September 1st 2022 and May 31st 2023 were available for high-throughput sequencing (HTS). These isolates represent eight *emm* types (Supplementary Table S1), with the majority being *emm*1 or *emm*12. To contextualize the *emm*1 (n=30) and *emm*12 (n=12) isolates sequenced for this study, the raw sequencing reads for 135 *emm*1 non-invasive isolates recovered in the United Kingdom [1], 99 *emm*1 and 239 *emm*12 invasive and non-invasive isolates from Denmark [2] and 64 *emm*12 isolates from a diverse collection were downloaded [3] from the European Nucleotide Archive (ENA). Additionally, 5 *emm*12 genome assemblies and the complete genome of M1 reference strain MGAS5005 were downloaded from the NCBI RefSeq database to include in the data sets.

High-throughput sequencing

Genomic DNA was extracted from cultures of GAS grown overnight in Todd-Hewitt broth (Oxoid, Basingstoke, UK) using the PureLink genomic DNA minikit (Invitrogen, Carlsbad, CA, USA). The initial bacterial lysis step was carried out in the presence of 45 U of mutanolysin (Sigma-Aldrich, St. Louis, MO, USA) and 86 µg of hyaluronidase (Sigma-Aldrich, St. Louis, MO, USA). Whole-genome sequencing libraries were generated using the Nextera DNA library preparation kit (Illumina, San Diego, CA, USA). The libraries were sequenced in an Illumina NextSeq 550 or NextSeq 2000 instrument.

Sequencing data analysis

Raw sequence reads were assembled with INNUca v4.2.3 [4] with the following parameters: --speciesExpected Streptococcus pyogenes, --genomeSizeExpectedMb 2, --runInsertSize, --maxNumberContigs 300, --trueCoverageProceed, and --fastQCproceed. All samples passed the quality control steps related to sequence quality or assembly coverage.
Genome annotation was performed with Prokka 1.14.6 [5] with the following parameters: --addgenes, --usegenus, --rfam, --rnammer, --increment 10, --mincontiglen 1, --gcode 11 and --kingdom Bacteria.
In silico Sequence Type (ST) prediction was performed using MLST v2.23.0 [6] with default parameters and the PubMLST database available at https://pubmlst.org/organisms/streptococcus-pyogenes/.
The emm type was determined from the draft genomes using emmtyper v0.2.0 [7] with verbose mode and the database available at https://www2.cdc.gov/vaccines/biotech/strepblast.asp.
The presence of the SNPs previously associated with the $M1_{UK}$ and the $M1_{DK}$ lineages was determined from the draft genomes using Snippy v4.6.0 [8] with the following parameters: --mincov 2 and --minqual 0. The complete genome of M1 reference strain MGAS5005 (RefSeq accession no. GCF_000011765.3) was used as reference.
The presence of antimicrobial resistance conferring genes and the *speC* gene was determined using ABRicate [9] with default parameters and the NCBI and VFDB databases, respectively.

The *parC* and *gyrB* genes were visually inspected for changes in the quinolone resistance determining region (QRDR) relative to the most frequent allele in the quinolone susceptible isolates using Geneious Prime 2022.

cgMLST analysis

The allelic profiles of the 265 *emm*1 and 320 *emm*12 genomes were determined with chewBBACA v3.2.0 [10] using default parameters and the *Streptococcus pyogenes* wgMLST schema available in the Chewie-NS platform [11] (https://chewbbaca.online/species/1/schemas/1).
Allelic profiles of the core loci (shared by 100% of the isolates under analysis) were used to create minimum spanning trees with the goeBURST algorithm in the online version of PHYLOViZ [12, 13].

Phylogenetic analysis

The multiple sequence alignment (MSA) for the translated core loci in the 265 *emm*1 (1,249 loci, 395,067 amino acids) and 320 *emm*12 (1,128 loci, 356,221 amino acids) genomes was computed with MAFFT v7.520 [14]. The approximately-maximum-likelihood phylogenetic trees were inferred based on the MSA of the translated core loci with FastTree v2.1.11 [15] using default parameters. Phylogenetic trees and the associated metadata were visualized and exported using the Interactive Tree Of Life platform [16].

Supplemental Figures

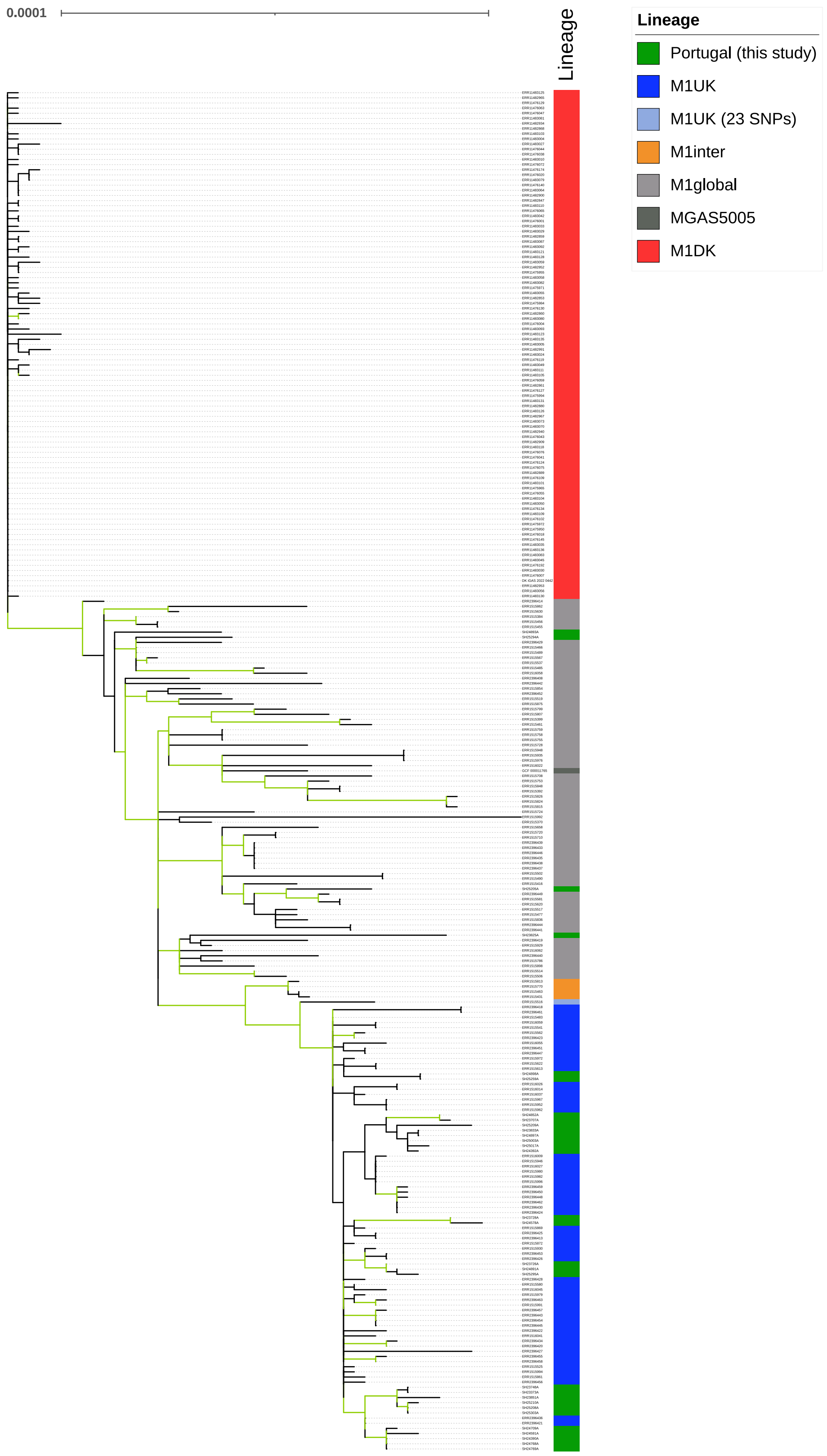

Figure S1 - Approximately-maximum-likelihood phylogenetic tree of 265 GAS emm1 isolates based on the MSA of 1,249 core loci. Splits colored in green indicate >0.9 Shimodaira-Hasegawa support values. The lineage of each isolate is indicated on the color strip.

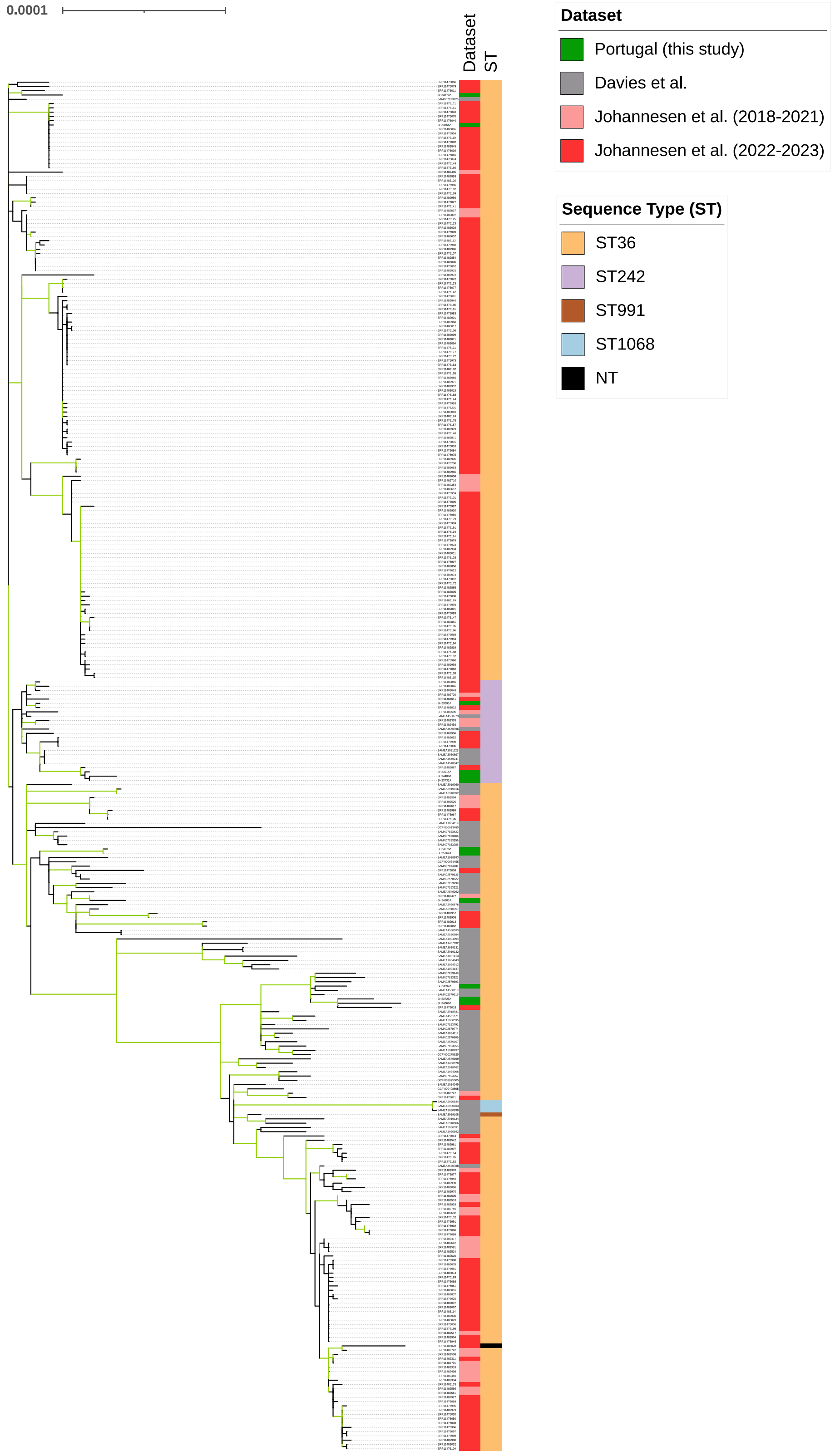

Figure S2 - Approximately-maximum-likelihood phylogenetic tree of 320 GAS emm12 isolates based on the MSA of 1,128 core loci. Splits colored in green indicate >0.9 Shimodaira-Hasegawa support values. The dataset and ST of each isolate is indicated on the color strips.

Supplementary References

1. Lynskey NN, Jauneikaite E, Li HK, Zhi X, Turner CE, Mosavie M, et al. Emergence of dominant toxigenic M1T1 *Streptococcus pyogenes* clone during increased scarlet fever activity in England: a population-based molecular epidemiological study. Lancet Infect Dis. 2019 Nov;19(11):1209–18.

2. Johannesen TB, Munkstrup C, Edslev SM, Baig S, Nielsen S, Funk T, et al. Increase in invasive group A streptococcal infections and emergence of novel, rapidly expanding sub-lineage of the virulent *Streptococcus pyogenes* M1 clone, Denmark, 2023. Eurosurveillance. 2023 Jun 29;28(26).

3. Davies MR, McIntyre L, Mutreja A, Lacey JA, Lees JA, Towers RJ, et al. Atlas of group A streptococcal vaccine candidates compiled using large-scale comparative genomics. Nat Genet. 2019 Jun 27;51(6):1035–43.

4. Bioinformatics @ Molecular Microbiology and Infection Unit. B-UMMI/INNUca [Internet]. GitHub. 2019 [cited 2023 Aug 31]. Available from: https://github.com/B-UMMI/INNUca

5. Seemann T. Prokka: rapid prokaryotic genome annotation. Bioinformatics. 2014 Jul 15;30(14):2068–9.

6. Seemann T. mlst [Internet]. Github. 2022 [cited 2023 Aug 31]. Available from: https://github.com/tseemann/mlst

7. Microbiological Diagnostic Unit Public Health Laboratory. emmtyper - Emm Automatic Isolate Labeller [Internet]. GitHub. 2023 [cited 2023 Aug 31]. Available from: https://github.com/MDU-PHL/emmtyper

8. Seemann T. Snippy [Internet]. Github. 2020 [cited 2023 Aug 31]. Available from: https://github.com/tseemann/snippy

9. Seemann T. ABRicate - Mass screening of contigs for antimicrobial and virulence genes [Internet]. Github. 2020 [cited 2023 Aug 31]. Available from: https://github.com/tseemann/abricate

10. Silva M, Machado MP, Silva DN, Rossi M, Moran-Gilad J, Santos S, et al. chewBBACA: A complete suite for gene-by-gene schema creation and strain identification. Microb Genomics. 2018 Mar 1;4(3).

11. Mamede R, Vila-Cerqueira P, Silva M, Carriço JA, Ramirez M. Chewie Nomenclature Server (chewie-NS): a deployable nomenclature server for easy sharing of core and whole genome MLST schemas. Nucleic Acids Res. 2021 Jan 8;49(D1):D660–6.

12. Nascimento M, Sousa A, Ramirez M, Francisco AP, Carriço JA, Vaz C. PHYLOViZ 2.0: providing scalable data integration and visualization for multiple phylogenetic inference methods. Kelso J, editor. Bioinformatics. 2017 Jan 1;33(1):128–9.

13. Ribeiro-Gonçalves B, Francisco AP, Vaz C, Ramirez M, Carriço JA. PHYLOViZ Online: web-based tool for visualization, phylogenetic inference, analysis and sharing of minimum spanning trees. Nucleic Acids Res. 2016 Jul 8;44(W1):W246–51.

14. Katoh K, Standley DM. MAFFT Multiple Sequence Alignment Software Version 7: Improvements in Performance and Usability. Mol Biol Evol. 2013 Apr 1;30(4):772–80.

15. Price MN, Dehal PS, Arkin AP. FastTree 2 – Approximately Maximum-Likelihood Trees for Large Alignments. Poon AFY, editor. PLoS One. 2010 Mar 10;5(3):e9490.

16. Letunic I, Bork P. Interactive Tree Of Life (iTOL) v5: an online tool for phylogenetic tree display and annotation. Nucleic Acids Res. 2021 Jul 2;49(W1):W293–6.
